# Changes in Index of Microcirculatory Resistance during PCI in the Left Anterior Descending Coronary Artery in Relation to Total Length of Implanted Stents

**DOI:** 10.1155/2019/1397895

**Published:** 2019-12-01

**Authors:** Christina Ekenbäck, Fadi Jokhaji, Nikolaos Östlund-Papadogeorgos, Habib Mir-Akbari, Rikard Linder, Nils Witt, Mattias Törnerud, Bassem Samad, Jonas Persson

**Affiliations:** ^1^Division of Cardiovascular Medicine, Department of Clinical Sciences, Karolinska Institutet, Danderyd University Hospital, Stockholm, Sweden; ^2^Department of Clinical Science and Education, Karolinska Institutet, Unit of Cardiology, Södersjukhuset, Stockholm, Sweden

## Abstract

**Aim:**

To investigate the relationship between stent length and changes in microvascular resistance during PCI in stable coronary artery disease (CAD).

**Methods and Results:**

We measured fractional flow reserve (FFR), index of microcirculatory resistance (IMR), and coronary flow reserve (CFR) before and after stenting in 42 consecutive subjects with stable coronary artery undergoing PCI with stent in the LAD. Patients that had very long stent length (38–78 mm) had lower FFR before stenting than patients that had long (23–37 mm) and moderate (12–22 mm) stent length (0.59 (±0.16), 0.70 (±0.12), and 0.75 (±0.07); *p*=0.002). FFR improved after stenting and more so in subjects with very long stent length compared to long and moderate stent length (0.27 (s.d ± 16), 0.15 (s.d ± 0.12), and 0.12 (s.d ± 0.07); *p* for interaction = 0.013). Corrected IMR (IMR_corr_) increased after stenting in subjects who had very long stent length, whereas IMR_corr_ was lower after stenting in subjects who had long or moderate stent length (4.6 (s.d. ± 10.7), −1.4 (s.d. ± 9,9), and −4.2 (s.d. ± 7.8); *p* for interaction = 0.009).

**Conclusions:**

Changes in IMR during PCI in the LAD in stable CAD seem to be related to total length of stents implanted, possibly influencing post-PCI FFR. Larger studies are needed to confirm the relationship.

## 1. Introduction

Fractional flow reserve (FFR) is a validated and reproducible measure of the functional severity of a coronary artery lesion [1, 2]. FFR-guided PCI with DES and optimal medical therapy (OMT) improves outcome and angina symptoms for patients with stable coronary artery disease compared to OMT alone [3, 4]. Furthermore, higher post-PCI FFR is associated with better outcome [5, 6]. However, increased coronary microvascular resistance is associated with higher post-PCI FFR [7], and coronary microvascular dysfunction (CMVD) is associated with long-term recurrence of restenosis [8].

Index of microcirculatory resistance (IMR) is a validated measurement of coronary microvascular resistance [9], and high IMR (>25 units) can be considered to be a proxy for CMVD [10]. IMR is derived from thermodilution measurements at peak hyperaemia [9], thereby eliminating the variability of coronary resting flow. Importantly, IMR is measured simultaneously with FFR [11]; thus, the physiological significance of epicardial lesions and the coronary microcirculatory function can be concurrently assessed.

Whether epicardial stenosis affects hyperaemic coronary microvascular resistance is debated and data are conflicting. Studies have shown that PCI lowers microvascular resistance immediately after stenting [12–14], whereas other reports have shown that microvascular resistance is independent of epicardial lesions [15–18]. The relationship between stent length and changes in microvascular resistance is not known. We hypothesised that there was no improvement, or even impairment, in microvascular resistance post-PCI with very long total stent length. For this purpose, we measured FFR, IMR, and coronary flow reserve (CFR) with thermodilution before and after stenting in 42 consecutive subjects with stable coronary artery undergoing PCI with stent of the LAD. The relationships between changes in coronary flow indices and total stent length were analysed.

## 2. Methods

### 2.1. Subjects

Consecutive patients, 18–85 years of age, with an life expectancy of >2 years that were planned for coronary angiography due to stable coronary artery disease were included between 2^nd^ of September 2015 and 20^th^ of September 2017. Subjects were included before coronary anatomy was known. Patients with acute coronary syndrome, congestive heart failure with ejection fraction <39%, prior heart transplantation, prior coronary artery by-pass grafting, hypertrophic cardiomyopathy, valvular heart disease scheduled for surgery/intervention, cancer within three years of admission, peri- and/or myocarditis, contrast allergy, atrial fibrillation with ventricular rate >120, asthma bronchiale, and atrioventricular block II-III were excluded. After coronary angiography, measurements of FFR, CFR, and IMR in the LAD were conducted irrespectively of coronary anatomy. Subjects with chronic total occlusion in the LAD, heavy calcification, or tortuosity in the left main/LAD hindering safe passage of a guide-wire with pressure and temperature sensors were excluded at the discretion of the PCI operator. In total, 274 subjects were included after oral and written informed consent was obtained. In the current analysis, we report pre- to post-PCI changes in FFR, CFR, and IMR in relation to total stent length and other variables in subjects treated with PCI in the LAD who had flow indices measured both before and after PCI (*n* = 42). Ethical permission was approved by the Regional Ethical Review Board in Stockholm (no 2015/962–31).

### 2.2. Flow Measurements

FFR, IMR, and CFR were measured in the LAD after initial coronary angiography and again after PCI of the LAD. A coronary guide-wire with pressure and temperature sensors (Pressure Wire X; Abbott Inc., CA, USA) was advanced in the LAD (sensors >70 mm from the catheter tip and distal to the lesion). Three millilitres of cold saline was injected into the LAD, and thermodilution curves were recorded. The procedure was repeated in total three times calculating the mean transit time (Tmn_base_). Then, adenosine (dosage of 167 *μ*g/kg/min) was administered intravenously in a large cubital vein for two minutes to induce stable hyperaemia. A higher infusion rate of adenosine 1 mg/ml than in other studies [19, 20] was used for feasibility (70 kg patient receives an infusion of 700 ml per hour, 80 kg patient receives an infusion of 800 ml per hour, and so on.). Furthermore, infusion rates of 160–180 *μ*g/kg/min are safe and recommended to receive maximal hyperaemia [20, 21]. Again, three millilitres of cold saline was injected in the LAD three times, and hyperaemic thermodilution curves (Tmn_hyp_) and distal (Pd) and proximal (Pa) coronary pressure were recorded. Measurements were repeated after PCI in the LAD in the same position as before stenting.

FFR was calculated by dividing Pd by Pa (Pd/Pa) during hyperaemia. CFR was calculated as the ratio between Tmn_base_ and Tmn_hyp_ (Tmn_base_/Tmn_hyp_). IMR was calculated as the product of Tmn_hyp_ and Pd (Tmn_hyp_ × Pd). IMR can be overestimated in subjects with FFR ≤0.80 due to collateral flow, and thus corrected IMR (IMR_corr_) was calculated according to Yong [22]: IMR_corr_ = Pa × Tmn_hyp_ × ([1.35 × Pd/Pa] − 0.32).

### 2.3. Statistics

Median values and interquartile range (IQR) are presented for continuous variables. Numbers and proportions are presented for categorical variables. Stent length was categorized into three different categories since the variable is ordinal and non‐normally distributed; moderate (12–22 mm), long (23–37 mm), and very long (38–78 mm) stent length. Linear regression was used to compare flow indices between groups. Two-way repeated measures ANOVA analyses (Wilks-Lambda) were used to analyse changes in IMR and CFR before and after stenting in relation to stent length group. Non‐normally distributed variables, including IMR_corr_, were log-transformed before analysis. *α* was two-sided and set to 0.05. IBM SPSS Statistics 23.0 was used for analyses.

## 3. Results

### 3.1. Baseline Procedural Characteristics

Forty-two patients underwent stenting of the LAD and had flow indices measured before and after stenting. Seven patients were women, seven had diabetes mellitus, and two thirds of the patients had one-vessel disease (Table 1). A majority (90%) of the patients had Canadian Cardiovascular Society grade I and II angina pectoris. All patients were stented with second generation drug-eluting stent (everolimus, *n* = 23; zotarolimus, *n* = 13; sirolimus, *n* = 6). One patient in the long stent length group (23–37 mm) received a biodegradable vascular scaffold, and one patient in the very long stent length group (38–78 mm) was stented also in the left main. All patients were on double antiplatelet therapy. Two patients were on ticagrelor, of which one was a non‐responder to clopidogrel (Multiplate® ADPtest) and the other patient was allergic to clopidogrel. A few (*n* = 4) lesions were treated by direct stenting, a majority of the stents were post-dilated (Table 2), and no debulking devices such as rotational atherectomy were used. Patients that had very long stent length had lower FFR before stenting and higher Tmn_hyp_ after stenting than the other two groups (Table 3).

### 3.2. Changes in Flow Indices

**Figure 1:**
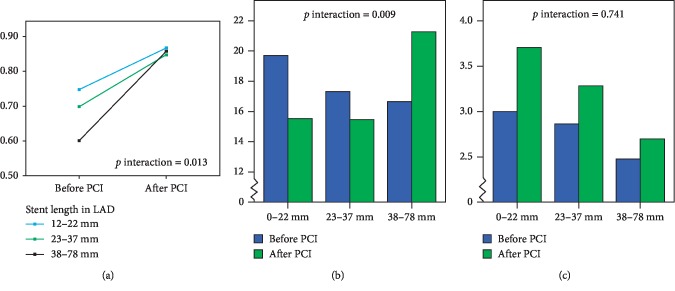
Changes in FFR (a), IMR_corr_ (b), and CFR (c) before and after PCI in relation to total stent length in LAD.

As expected, FFR was improved after stenting and more so in subjects with very long stent length (*p* for interaction = 0.013; Figure 1(a)). Changes in IMR_corr_ were related to stent length in LAD; IMR_corr_ increased after stenting in subjects who had very long stent length (38–78 mm), whereas IMR_corr_ was attenuated after stenting in subjects who had moderate or long (12–22 mm and 23–37 mm, respectively) stent length (*p* for interaction = 0.009; Figure 1(b)). Changes in CFR were not related to stent length (Figure 1(c)).

## 4. Discussion

### 4.1. Implications

Our data shows that PCI with very long stent length (≥38 mm) in the LAD is associated with larger improvement in FFR than in moderate or long stent length. The improvement in FFR was accompanied by a rise in IMR_corr_ after stenting for very long stent length, whereas moderate and long stent length was associated with attenuated IMR_corr_.

The findings have two important implications: (i) PCI with very long total stent length in patients with stable coronary artery disease in the elective setting is associated with a rise in resistance after PCI in the coronary microcirculation and (ii) improved FFR values after PCI with very long total stent length may be partly explained by the immediate rise in the resistance of the coronary microcirculation after the procedure (on average 4.6 units IMR_corr_-change from pre- to post-PCI).

Total stent length can be considered to be a proxy for both atherosclerotic burden and complexity of PCI procedures. In more complex PCI with very long total stent length, more balloon dilatations, and interactions with a larger part of the vessel wall, the occurrence of impaired microcirculation and rise in the resistance are likely more frequent than in less complex PCI, which explain our findings with elevated IMR after PCI with very long stent length. Supporting this, is a randomized trial of direct stenting compared to stenting with predilatation (more complex technique), indicating that direct stenting with fewer dilatations is less traumatic for the coronary microcirculation as compared to conventional stenting [23]. Elective PCI might impair resting microvascular perfusion [24] through several different mechanisms acutely elevating the coronary microcirculatory resistance; release of local vasoconstrictors [25], vasoconstriction [26], impairment of endothelial function [27, 28], and distal embolization [29, 30]. Microvascular impairment is more likely to occur during more complex PCI and/or in very long lesions than in less complex interventions.

Coronary microvascular disease is one of the limitations when identifying ischemic lesions with FFR [21]. It is difficult to appreciate the effects of immediate (and possibly transient) acute rise in coronary microcirculation resistance corresponding to 4.6 units IMR_corr_ on distal pressure in the LAD (P_d_) and subsequent elevation of FFR values after PCI. Studies have consistently shown that higher FFR values after stenting is associated with lower MACE rates [5, 6, 31, 32]. A recent meta-analysis of post-PCI FFR measurements in FAME-1 and FAME-2 showed that the best cut-off value for predicting two-year vessel-oriented composite endpoint (death, MI, revascularization) was 0.915, with a sensitivity of 75% and specificity of 43%. However, the authors conclude that its predictive value is too low for optimization of PCI procedures [32]. Assessing FFR in combination with post-PCI IMR provides information about *the* treated epicardial lesion and the microcirculatory function immediately after the intervention. This might improve sensitivity, specificity, and predictive value for future major adverse cardiovascular events. The interpretation of post-PCI FFR may benefit from concurrent assessment of the coronary microcirculation, but it needs to be evaluated in studies.

Pre-PCI IMR is associated with post-PCI in a several studies [12, 33, 34]. In our study, pre-PCI IMR are accounted for in the analysis since we are studying changes in IMR during PCI. Diabetes mellitus is associated with post-PCI IMR in a small study mixing acute coronary syndromes and stable coronary artery disease [33]. Diabetes mellitus was not an interaction term for changes in IMR in our study (data not shown), possibly due to low power with only seven subjects with diabetes mellitus.

In experimental human and animal studies, assessment of IMR is independent of artificial stenosis created by balloons and a vascular occluder [17, 18], and studies of IMR before and after PCI have shown that there are no alterations in microvascular resistance when collateral flow is taken into account [15, 16]. However, other studies have shown that microvascular resistance is attenuated (improved) immediately after PCI [12–14, 34], and these effects seem to persist after 10 months of follow-up [12]. Large studies are needed to evaluate the natural course of immediate rise of microcirculatory resistance after stenting, as is the case with subjects with very long total stent length in our study.

We do know that total stent length is associated with restenosis [35], but the contribution of CMVD in restenosis is not established. In one study of 29 patients, lower coronary blood flow response to cold-pressor test measured by transthoracic Doppler echocardiography (a proxy for CMVD) 24 hours after stent implantation in the LAD was associated with long-term recurrence of restenosis [8], indicating that acute coronary microvascular function is important in the pathogenesis of restenosis. Large studies are needed to establish the role of post-PCI CMVD in stable coronary artery disease in relation to long-term prognosis.

### 4.2. Limitations

This is an observational study and conclusions about causal effects cannot be made. The number of observations is small, and lack of association of IMR and/or changes in IMR with procedural or baseline characteristics variables could be secondary to low power. The results are confined to the LAD only, and effects in the left circumflex or the right coronary artery are not examined. We have not routinely measured cardiac markers after PCI, and thus we cannot present the frequency of type 4a myocardial infarction. Assessment of IMR directly after stenting does not reveal if alterations of post-PCI IMR are transient or permanent.

There are also strengths in this study; all patients in our study had stable coronary artery disease only, and the flow was assessed before and after PCI in the LAD for all patients. The patients were all included prospectively before the coronary anatomy was known which diminishes the inclusion bias inflicted by the perception of the PCI operators.

## 5. Conclusions

Changes in IMR during PCI in the LAD in stable CAD seem to be related to total length of stents implanted, possibly influencing post-PCI FFR. In this study, PCI with very long total stent length was associated with an immediate rise in coronary microvascular resistance. Larger studies are needed to confirm the relationship between total stent length and changes in IMR during PCI.

## Figures and Tables

**Table 1:** Baseline characteristics.

Age, years (s.d.)	65.3 (8.2)
Female gender, *n* (%)	7 (17)
Diabetes mellitus, *n* (%)	7 (17)
Previous myocardial infarction, *n* (%)	5 (12)
Previous PCI, *n* (%)	9 (21)
Atrial flutter, *n* (%)	2 (5)

Smoking, *n* (%)	
Never	24 (57)
Previous	13 (31)
Smoker	5 (12)

Canadian Cardiovascular Society grade, *n* (%)	
I	15 (36)
II	23 (55)
III	3 (7)
IV	1 (2)

Body mass index (kg/m^2^), median (IQR)	27 (24–30)
Total cholesterol (mmol/L), median (IQR)	4.3 (3.7–5.1)
Low density lipoprotein cholesterol (mmol), median (IQR)	2.4 (1.9–3.1)
HbA_1_c (mmol/mol), median (IQR)	39 (35–42)
Creatinine clearance (mL/min/1.73 m^2^), median (IQR)	82 (69–96)

Medication at inclusion	
Aspirin	42 (100)
Clopidogrel	40 (95)
Ticagrelor	2 (5)
AII-receptor antagonists, *n* (%)	12 (29)
Beta-blockers, *n* (%)	26 (62)
Nitrates, *n* (%)	10 (24)
Calcium antagonists, *n* (%)	13 (31)
ACE inhibitors, *n* (%)	9 (21)
Lipid-lowering therapy at admission	32 (76)

IQR = interquartile range.

**Table 2:** Procedural characteristics.

		Total stent length in LAD		
		12–22 mm	23–37 mm	38–78 mm
		*n* = 14	*n* = 14	*n* = 14
Number of stents in LAD	1	14	9	5
	2	0	5	7
	3	0	0	2

Direct stenting (no predilatation)	Yes	1	3	0
	No	13	9	13

Post dilatation	Yes	7	9	14
	No	7	5	0

Largest stent in LAD (mm), median (IQR)		3.5 (3–3.5)	3.5 (3.5–3.5)	3.5 (3–3.5)
Smallest stent in LAD (mm), median (IQR)		3.5 (3–3.5)	3.5 (3–3.5)	3 (2.75–3.5)
Total stent length in LAD (mm), median (IQR)		20 (16–20)	29 (26–34)	46 (38–54)
Maximum dilatation pressure, median (IQR)		20 (16–20)	20 (16–20)	20 (20–20)

IQR = interquartile range.

**Table 3:** Flow indices in relation to total stent length in LAD.

	Total stent length in LAD			
	12–22 mm *n* = 14	23–37 mm *n* = 14	38–78 mm *n* = 14	*p* ^*∗*^
Before PCI				
Pa hyperaemia (mm Hg)	82 (65–84)	76 (61–87)	72 (65–88)	0.903
Pd hyperaemia (mm Hg)	58 (45–65)	50.5 (40–67)	49 (41–56)	0.242
FFR	0.77 (0.69–0.80)	0.74 (0.7–0.77)	0.66 (0.46–0.74)	0.002
IMR^†^	20 (11–27)	16 (9–26)	16 (10–20)	0.557
IMR_corr_^†^	19 (10–25)	14 (8–24)	14 (10–18)	0.329
CFR	2.95 (2.5–3.8)	2.8 (1.5–4.1)	2.15 (1.2–3.8)	0.305
T_mn_ baseline (seconds)^†^	1.0 (0.65–1.49)	0.72 (0.47–1.37)	0.89 (0.63–1.13)	0.305
T_mn_ hyperaemia (seconds)^†^	0.32 (0.23–0.54)	0.44 (0.2–0.5)	0.32 (0.24–0.42)	0.750

After PCI				
Pa hyperaemia (mm Hg)	72 (64–81)	71 (57–84)	72 (65–80)	0.794
Pd hyperaemia (mm Hg)	64 (55–69)	62 (48–73)	65 (52–72)	0.884
FFR	0.86 (0.84–0.9)	0.84 (0.82–0.87)	0.86 (0.8–0.9)	0.660
IMR^†^	14 (10–17)	15 (9–23)	22 (14–26)	0.118
IMR_corr_^†^	13 (10–17)	14 (9–22)	21 (13–25)	0.060
CFR	3.6 (2.5–4.9)	3.0 (2–4.7)	2.3 (1.5–4.0)	0.072
T_mn_ baseline (seconds)^†^	0.85 (0.58–1.31)	0.61 (0.55–0.88)	0.72 (0.52–1.22)	0.824
T_mn_ hyperaemia (seconds)^†^	0.20 (0.17–0.3)	0.21 (0.17–0.36)	0.32 (0.26–0.34)	0.045

Median (interquartile range); CFR = coronary flow reserve; FFR = fractional flow reserve; IMR = index of microcirculatory resistance; IMR_corr_ = corrected IMR; Pa = arterial pressure during hyperaemia; Pd = distal pressure during hyperaemia; *T*_mn_ = mean transit time; ^*∗*^linear regression; ^†^variables log-transformed before analysis.

## Data Availability

The data used to support the findings of this study are available from the corresponding author upon request.

## Abbreviations

FFR: Fractional flow reserve
OMT: Optimal medical therapy
CMVD: Coronary microvascular dysfunction
IMR: Index of microcirculatory resistance
IMR_corr_: Corrected IMR
CFR: Coronary flow reserve
T_mn_: Mean transit time
P_a_: Proximal coronary pressure
P_d_: Distal coronary pressure

## Additional Points

*Impact on Daily Practice*. Complex PCI with very long total stent length in the LAD is associated with acute rise in microcirculatory resistance in subjects with stable coronary artery disease. Acute rise in coronary microcirculatory resistance might augment FFR and thus compromise the interpretation of post-PCI FFR.
